# Evaluating Population Genetic Structure and Demographic History of *Quercus spinosa* (Fagaceae) Based on Specific Length Amplified Fragment Sequencing

**DOI:** 10.3389/fgene.2019.00965

**Published:** 2019-10-03

**Authors:** Miao-Miao Ju, Li Feng, Jia Yang, Yan-Ci Yang, Xiao-Dan Chen, Gui-Fang Zhao

**Affiliations:** ^1^Key Laboratory of Resource Biology and Biotechnology in Western China (Ministry of Education), College of Life Sciences, Northwest University, Xi’an, China; ^2^School of Pharmacy, Xi’an Jiaotong University, Xi’an, China; ^3^School of Biological Science and Technology, Baotou Teachers’ College, Baotou, China

**Keywords:** *Quercus spinosa*, SLAF-seq, population structure, ecological niche model, demographic modeling

## Abstract

Effectively identifying the genetic structure and related factors of a species can facilitate understanding the evolutionary history of the species. Phylogeographic patterns and genetic data are essential in investigating the species historical processes and diversification that response to environmental, climatic and geological influences. In this study, Specific Length Amplified Fragment Sequencing (SLAF-seq) data and ecological niche models (ENMs) are combined to identify the genetic structure and demographic modeling of *Quercus spinosa*, and evaluate the impacts of historical range shifts, climatic variation, and landscape factors on this species. The population topology and genetic divergence of the Cenozoic were inferred by a site frequency spectrum based composite-likelihood approach which is a novel strategy for maximizing the utility of linked SLAF markers. The overall genetic structure using model-based and model-free clustering methods was consistently identified as two geographically distinct genetic clusters. A deep divergence between two natural lineages (i.e., a western Himalaya-Hengduan Mountains lineage and an eastern Qin-ling Mountains lineage) was observed. The demographic modeling and Niche reconstruction indicated that the two groups were diverged in the late Miocene and then presented as two distinct genetic lineages. With the Quaternary glacial climate fluctuation, two groups had continuous asymmetrical secondary contact and gene exchange in the Sichuan Basin during the last glacial maximum. Besides, a significant relationship between genetic distance and geography in all individuals was identified by the Mantel test. Overall, this study 1) contributes to a better understanding of the role played by Quaternary climatic fluctuation in the present-day distributions of *Q. spinosa*; 2) provides a comprehensive view of the genome-wide variation of sclerophyllous forests in ecological adaptive evolution; 3) indicates that dispersal limitation and ecological divergence contribute to the genome-wide differentiation of *Q. spinosa*, which supports a hypothesis that complex geography and climatic changes strongly influence the evolutionary origin and history of the species.

## Introduction

The genetic diversity and population structure of species are largely determined by intrinsic traits, external zoology environment including anthropogenic activities and historical events (Avise, 2000). Phylogeographic studies showed that the population genetic structure and demographic history of extant species were profoundly shaped by past historical processes, such as geological and climatic events (Hewitt, 1996; Ye et al., 2017). The organisms could be affected by complex ecological factors in the natural habitat (Bailey et al., 2013; Xu, 2015). Environmental heterogeneity has the potential to maintain intraspecific divergence and acts as an important driver for generating biological diversity (Fan et al., 2013; Ju et al., 2018). The ecological differentiation through adaptation to local environment reflects a balance between natural selection and the homogenization effect of the gene flow (Slatkin, 1987; Garant et al., 2007).

Quaternary glaciation (about 2–3 million years ago) involved multiple successive cycles of glacial and interglacial events, which brought dramatic changes in temperature and precipitation on earth (Webb and Bartlein, 1992). The responses of plants to climate changes are characterized by the migration (i.e., change in geographical distribution) and adaptive evolution (i.e., the formation of new species) (Chen et al., 2011). The Quaternary glaciation was a major historical event that influenced the distribution pattern of extant species (Gong, 2007). Himalaya-Hengduan Mountains (HHM) (24°40′-34°00′N, 96°20′-104°30′E) located in Southwest China is a region with extreme elevation changes, and a corresponding diversity of habitats (Liu et al., 2013). Complex topography situation of HHM has weakened the impact of Quaternary glaciation fluctuation on organisms. Therefore, HHM is not only a vital glacial refugia of plants (Chen et al., 2011) but also a magnitude gene pool reserved in China. Meanwhile, Mt. Hengduan flora has a high biodiversity and becomes a harbor of numerous endemic alpine plants (Ying, 2001).

Through the application of modern molecular techniques and advanced bioinformatics analysis, including phylogeography and coalescent theory based analyses, a few studies revealed the genetic consequences of Quaternary climate change, and spatial pattern in species (Ribas et al., 2018; Silva et al., 2018). In China, phylogeographic studies mainly focused on the roles of historical orogenesis, climatic oscillations, and environmental heterogeneity in the evolutionary history of biotas in Qinghai-Tibet Plateau (QTP) and adjacent regions. The research object mostly focused on endangered species or herbaceous species with narrowing distribution areas (Liu et al., 2013; Ren et al., 2017); only few researches put emphasis on sclerophyllous forests (Shi et al., 2014; Wang et al., 2015). Though many great advancements and emerging patterns have been achieved in temperate species and a few tropical species, the genetic mechanism of evergreen species responses to the Quaternary glacial fluctuation in HHM should be further investigated. During the Late Miocene, the ancient Mediterranean retreated with the continuous uplift of the Himalayas, and the climate became cold and dry. The broad-leaved forests were declined and replaced by evergreen oak in this region (Zhou, 1993). The evergreen oak gradually became the dominant and constructive species with the characteristics of cold-proof and drought-enduring (Zhou, 1992; Zhou, 1999). Phylogeographical studies combined with molecular evidence indicated that continuous uplift of HHM from the late Miocene to early Pliocene accompanied by simultaneous cooling triggered the differentiation of *Quercus* sect. *Heterobalanus* (Zhou et al., 2003; Meng et al., 2017). Researches on evergreen oak showed that geological and climatic factors triggered the differentiation and evolution among three oak species in the Miocene (Feng et al., 2016), and past environmental changes in HHM may have catalyzed radiative diversifications in *Quercus quifolioides* (Du et al., 2017). However, the impact of orogenic activity and climatic fluctuation during recent geological history on evolutionarily stable taxa such as evergreen oaks are still not well understood.

*Quercus spinosa*, a member of the family Fagaceae, is an evergreen sclerophyll species. Geographically, *Q. spinosa* is widely distributed in the Himalaya-Hengduan Mountains (HHM), the Qin-ling Mountains, and the subtropical regions of Central China. *Q. spinosa* has the intrinsic traits of long generation times, large effective population sizes, and limited dispersal distance (Austerlitz et al., 2000), which is beneficial to reduce the impacts of glacial-interglacial climatic fluctuations on the genetic diversity and structure of organisms during the Quaternary. Thus, *Q. spinosa* is an ideal species for studying the effects of climate fluctuations and environmental changes on species diversification and evolution since Tertiary. In this study, the genome-wide variation patterns were characterized by SLAF-sequencing of the genome in *Q. spinosa*. Besides, a complementary approach combined with ecological niche modeling and demographic modeling is used to elucidate the divergence and demographic history of *Q. spinosa*. The main purpose of this research is to elucidate following specific questions: i) What is the population structure pattern of *Q. spinosa* within the whole genome? ii) Which differentiation model could be used to better explain the formation of the spatial distribution pattern of *Q. spinosa*? iii) Is there any evidence of local adaptation maintaining the genetic integrity of the species driven by geographical isolation and environmental factors?

## Materials and Methods

### Experimental Materials and DNA Extraction

A set of 130 *Q. spinosa* accessions from 38 wild populations, including all the relevant regions for the species that were described in Flora of China as a reference (Huang et al., 1999), were evaluated in the present study. All fresh young healthy leaves were dried in the silica gel in the field and the accessions were archived and stored in Northwest University (NWU). The distribution of sample sites was shown in **Supplementary Figure 1** and the detailed information including latitude and longitude of each sampled population was recorded in **Supplementary Table 1**.

The whole genomic DNA of leaf tissues was extracted *via* the CTAB method according to the manufacturer’s protocol (Chen et al., 2014). The DNA concentration and quality were detected by NanoDrop 2000 spectrophotometer (NanoDrop, Wilmington, DE, USA) and agarose gel electrophoresis, and the DNA samples were diluted to 18 ng μL^-1^ when it was necessary.

### High-Throughput Sequencing and Data Processing

Based on the fragment size and DNA concentration, the genomic DNA was selected for specific-locus amplified fragment (SLAF) library sequencing according to the procedures described by Sun with a few modifications (Sun et al., 2013). In order to enhance the efficiency of SLAF-seq, the enzymes and sizes of restriction fragments were evaluated by training data at first. The genomic DNA of the samples was digested with the *Rsa*I restriction enzyme, and the accuracy of the enzyme digestion experiment was evaluated by *Oryza sativa*. Then, the PCR amplification and purification ran out on 2% agarose gel. DNA fragments with 314–414bp (with indexes and adaptors) in size were isolated and diluted for paired-end sequencing on the Illumina HiseqTM 2500 sequencing platform (Illumina, Inc; San Diego, CA, USA) at Beijing Biomarker Technologies Corporation. The raw reads were processed for each sample using the software Dual-index (Kozich et al., 2013). After filtering out adapter reads, the sequencing quality was evaluated by calculating the guanine-cytosine (GC) content and Q30 value (i.e., Q_-score_ = -10×log_10_ *P*, indicating a 0.1% chance of an error and thus 99.9% confidence). In order to ensure the quality of sequencing, all paired-end reads were trimmed to 125bp×2 in length. These cleaned reads were stored in the NCBI SRA database (http://www.ncbi.nlm.nih.gov/sra/) under the BioProject ID PRJNA549662. Single nucleotide polymorphism (SNP) calling from multiple samples was performed using SAMtools (Li et al., 2009) and Genome Analysis Toolkit (GATK) software (McKenna et al., 2010). The parameters in the script were set by default, and only the loci in both software results of calling were kept. Then, the loci which were not polymorphic (minor allele frequency, MAF, of < = 0.05) were removed from the data set by VCFTOOLS (Danecek et al., 2011) and PLINK1.7 (Purcell et al., 2007). In addition, SNPs which exhibited significant deviation from Hardy-Weinberg (HWE, P < 10e^-6^) (Wigginton et al., 2005) were screened out, and the loci with extremely high heterozygosity resulting from false SNP calls or assembly errors were filtered. Finally, only the loci presented in all populations and successfully genotyped in at least 90% of individuals were kept and used in downstream analyses.

### Population Genetic Structure

Two individual-based approaches were used to investigate genetically differentiated populations in *Q. spinosa*. For hierarchical population-clustering analyses, the maximum-likelihood-based clustering algorithm was implemented in the program ADMIXTURE (Alexander et al., 2009) to determine the proportion of genetic ancestry of each individual from a specified number of ancestral populations (*K*) without *a priori* population designation. Then, a series of tests were conducted while the value of *K* was changed from 1 to 10, and accessions were assigned to a corresponding population based on their maximum membership probabilities. The most suitable *K* was determined by comparing threefold cross-validation (CV) error values across different values of *K* as described in the ADMIXTURE manual. For multivariate analyses, a discriminant analysis of principal components (DAPC) (Jombart and Ahmed, 2011) was conducted to identify genetic clusters of individuals first, which transformed individuals’ genotypes using a principal components analysis (PCA) (Pearson, 1901) prior and then identified maximize differentiation between groups (Jombart and Ahmed, 2011). The DAPC with the “adegenet” package (Jombart, 2008) was implemented in R studio (R Development Core Team, 2011). The optimal number of clusters was determined by running a principal components analysis and calculating the Bayesian information criterion (BIC) (Schawrz, 1978) for sequential *K*-values after the retention of 100 principal components. In order to avoid over-fitting of discriminant functions, an initial DAPC was used to find the α-score for each set of clusters which was used to select the number of principal components to retain in a subsequent re-analysis (Jombart, 2008; Jombart and Ahmed, 2011).

### Phylogenetic Tree and Mantel Test

In order to adopt a phylogenetic perspective, SNPs were used to generate maximum-likelihood trees. RAxML version 8.0.5 (Stamakis, 2014) was used for these analyses, and it implemented the GTRGAMMA model with ascertainment bias correction and a rapid bootstrap procedure with 100 replicates per run. Analyses were run with the entire SNP dataset which were considered for the clustering analyses. Trees were generated with ITOL (Interaction Tree of Life) on the website (http://itol.embl.de/). 

In order to evaluate the correlation analyses of the genetic similarity and geographic distances in the given populations, the genetic distance matrix among 38 *Q. spinosa* populations was calculated by the “ape” package in R studio (R Development Core Team, 2011), and based on a Mantel test (Mantel, 1967) with 999 matrix randomizations using GenAlEx version 6.501 (Peakall and Smouse, 2012).

### Demographic Modeling

In order to gain insight into the historical processes that generated the observed population structure, and estimate the timing of population splits and rates of gene flow among populations, the demographic models were introduced to the joint site frequency spectrum (SFS) of the SNP alignment in the program δaδi v1.7 (Gutenkunst et al., 2009). SFS-based methods are used for inferring divergence times, population tree topologies, and historical migration rates (Beichman et al., 2018), such as Lange and Pool (2018) and Laurent et al. (2016). For the demographic analyses, SLAF-seq VCF files were converted into δaδi’s input format by a custom R script according to Battey (Battey et al., 2018). According to the results of population structure, 2D analyses were carried out by a set of 32 demographic models (**Supplementary Table 3**) that represented different speciation scenarios including various timing and directionality of ancient or contemporary gene flow. Each model was run 10 times in the δaδi by an 80*90*100 grid space and the nonliner Broyden-Fletcher-Goldfarb-Shannon (BFGS) optimization routine. In order to calculate the best replicate run (highest composite likelihood) of each model, the model selection was conducted in an information-theoretic framework by Akaika Information Criterion (AIC) (Akaike, 1974).

### Ecological Niche Analyses

In order to verify whether the local adaptation could maintain the genetic structure of the species impelled by environmental factors, it is necessary to understand the climate niche of those plants and identify their suitable growing areas under current and future climate conditions. The geographical distribution of *Q. spinosa* across its whole range was registered in the Global Biodiversity Information Facility (GBIF, http://www.gbif.org) and the China Virtual Herbarium (CVH, http://www.cvh.ac.cn/) database. Google Earth (http://ditu.google.cn/) was used to determine the latitude and longitude when occurrence records lacked exact geo-coordinates. To avoid the impact of spatial autocorrelation on the results, the sample recorded the distance between latitude and longitude of which is below 0.4° were removed. In total, 211 unrepeatable geo-referenced occurrence records were collected.

Nineteen climatic variables of temperature and precipitation of the sampled locations were obtained from the WORLDCLIM database (http://www.worldclim.org/) at 2.5-min resolution. This coarse resolution could leave out relevant small-scale environmental heterogeneity, which may be relevant to maintain divergence. The species distribution model (SDM) in current climate conditions was inferred with the maximum entropy approach implemented in MAXENT 3.3 (Phillips et al., 2006), which just required geo-referenced occurrence data to predict the distribution of suitable habitat for a species coupled with environmental information for the whole study area (Elith et al., 2011). Model predictions were then checked against real observations through the area under the curve (AUC) of a receiver operating characteristic (ROC) plot. The conversion of the continuous suitability index maps into binary habitat and non-habitat charts required a probability threshold to determine potential changes in future habitat of *Q. spinosa*. Due to the promising feature of producing highly accurate predictions, the “maximum training sensitivity plus specificity” threshold was used to define habitat and non-habitat for *Q. spinosa*. For a proper evaluation, models were calibrated on 70% of the data and evaluated on the remaining 30%. Ten replicates were run, and the model performance was evaluated by the area under the curve (AUC) (Fielding and Bell, 1997) and true skill statistic (TSS) statistics (Allouche et al., 2006). The consensus model was then projected onto different past climatic periods by the data available in the WORLDCLIM dataset, i.e., the last interglacial (LIG; 0.14–0.12Ma) and the CCSM model of Last Glacial Maximum (LGM; 0.021–0.018Ma). All models can be found through http://www.worldclim.org/. 

## Results

### Sequences Data Quality and Processing

After the raw data was qualified, it could be further used for data mining and additional analyses. Across the 130 accessions examined in this study, a total of 304.69M reads were obtained. The reads length of samples ranged from 1,125,840 to 6,844,685 bp (**Supplementary Table 2**). All sequence passed the quality threshold and were used in the assembly of the SLAF-tags (**Supplementary Table 2**). The average of Q30 reached 91.73% (**Supplementary Table 2**) and the average of GC was 40.68% (**Supplementary Table 2**) among samples. The developed 104,457–212,997 SLAF loci with average depth was 10.16×. The sequences with the maximum depth of SLAF-tag were selected as reference sequences. Then, other sequences were blasted to the reference sequences by the BWA software, respectively (Li and Durbin, 2009). The GATK and SAMtools were used to develop the 124,607 high consistency SNPs for the following genetics analyses. Finally, 58,353 SNPs after filtration were identified by the PLINK software (maf 0.05; hwe 1×10^-6^) and used to investigate the genomic evolution of the population of 130 *Q. spinosa* accessions.

### Population Genetic Structure

**Figure 1:**
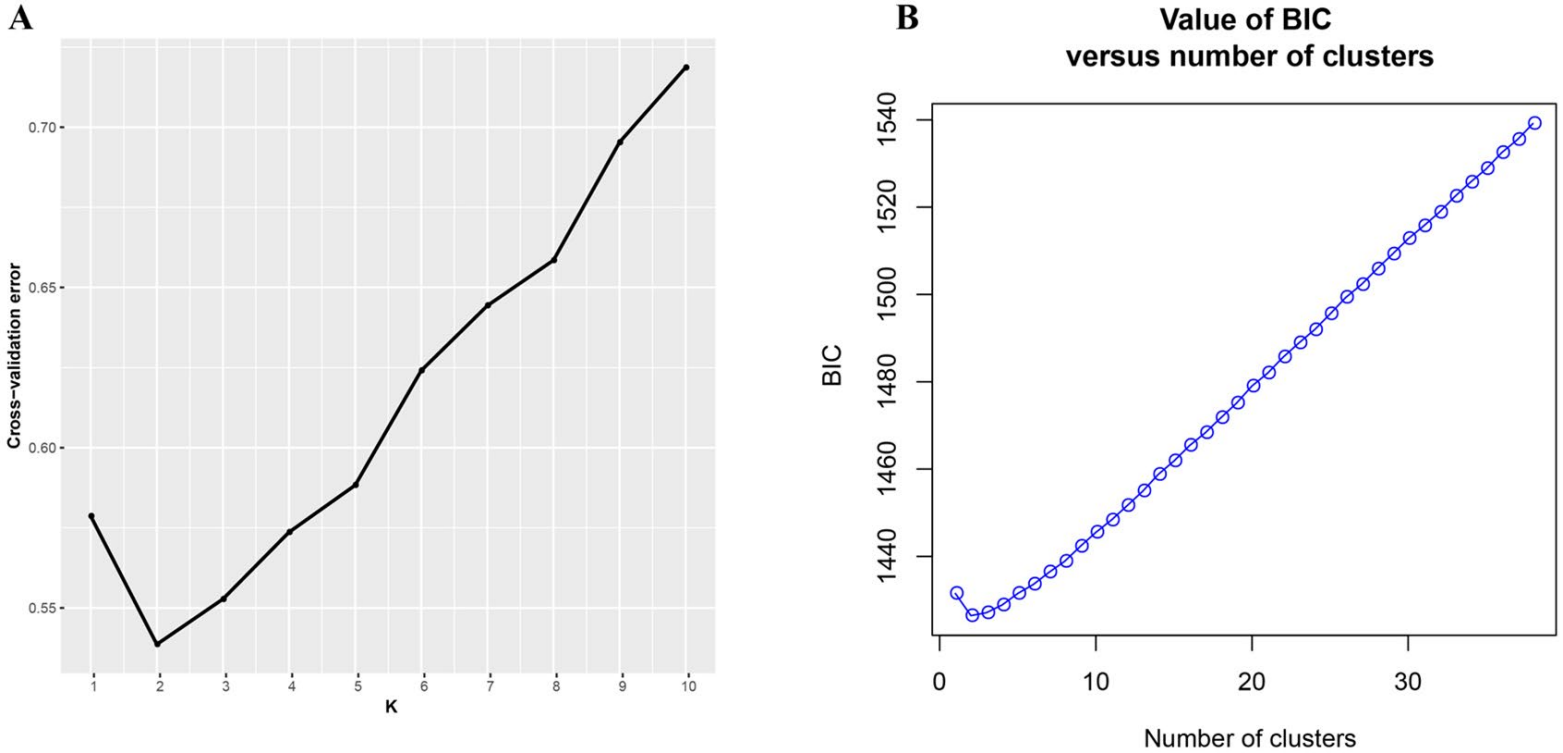
**(A)** Threefold cross-validation (CV) error values across different values of *K* in ADMIXTURE and **(B)** Bayesian information criterion for sequential K-values in DAPC.

ADMIXTURE results for the analysis of *Q. spinosa* indicated that a value of *K* = 2 was associated with the lowest cross-validation procedure (**Figure 1A**). The DAPC result was shown in **Figure 1B**. Likewise, the DAPC method chose *K* = 2 as the optimal number of genetic clusters to describe the data in this research. The ADMIXTURE and DAPC presented the evidence that a deep divergence was constructed. Two distinct lineages in the SNP dataset corresponded with *Q. spinosa* individuals.

**Figure 2:**
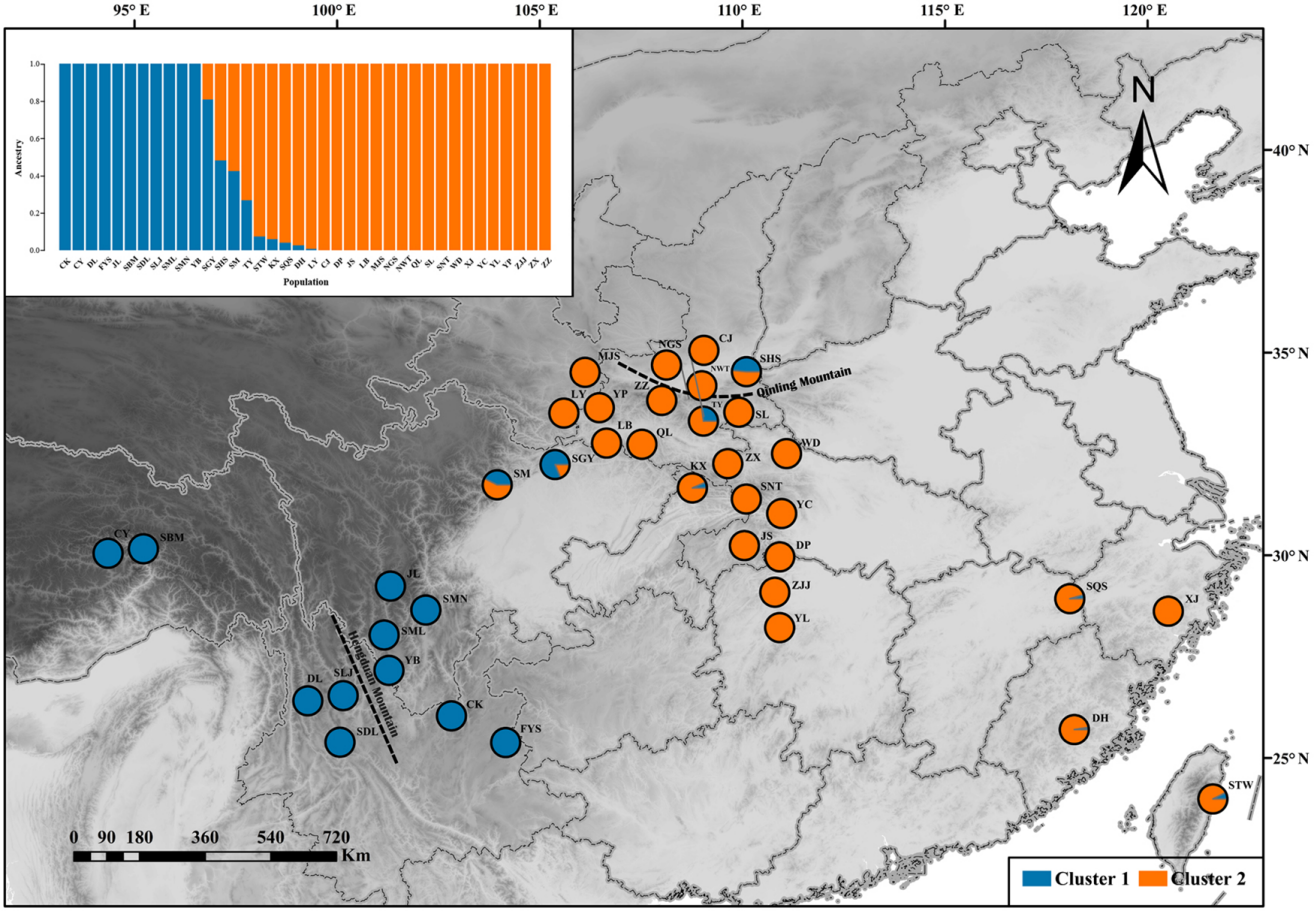
ADMIXTURE clustering analysis results for *Q. spinosa* populations based on their geographic distribution.

One hundred thirty individuals clustered into two groups, which were defined as “West” and “East”. The results of ADMIXTURE and DAPC showed that 13 populations clustered into an independent western group (cluster 1), and 25 populations clustered into another separate eastern group (cluster 2) (**Figure 2**). The transitional distribution of the two groups around the Sichuan basin formed the gene exchange. The results of clusters were in line with individuals’ geographical delimitation. Geographically, the 52 individuals from HHM clustered into the western group. Meanwhile, 78 individuals from Qin-ling Mountains and Central China clustered into the eastern group.

### Phylogenetic Tree and Mantel Test

**Figure 3:**
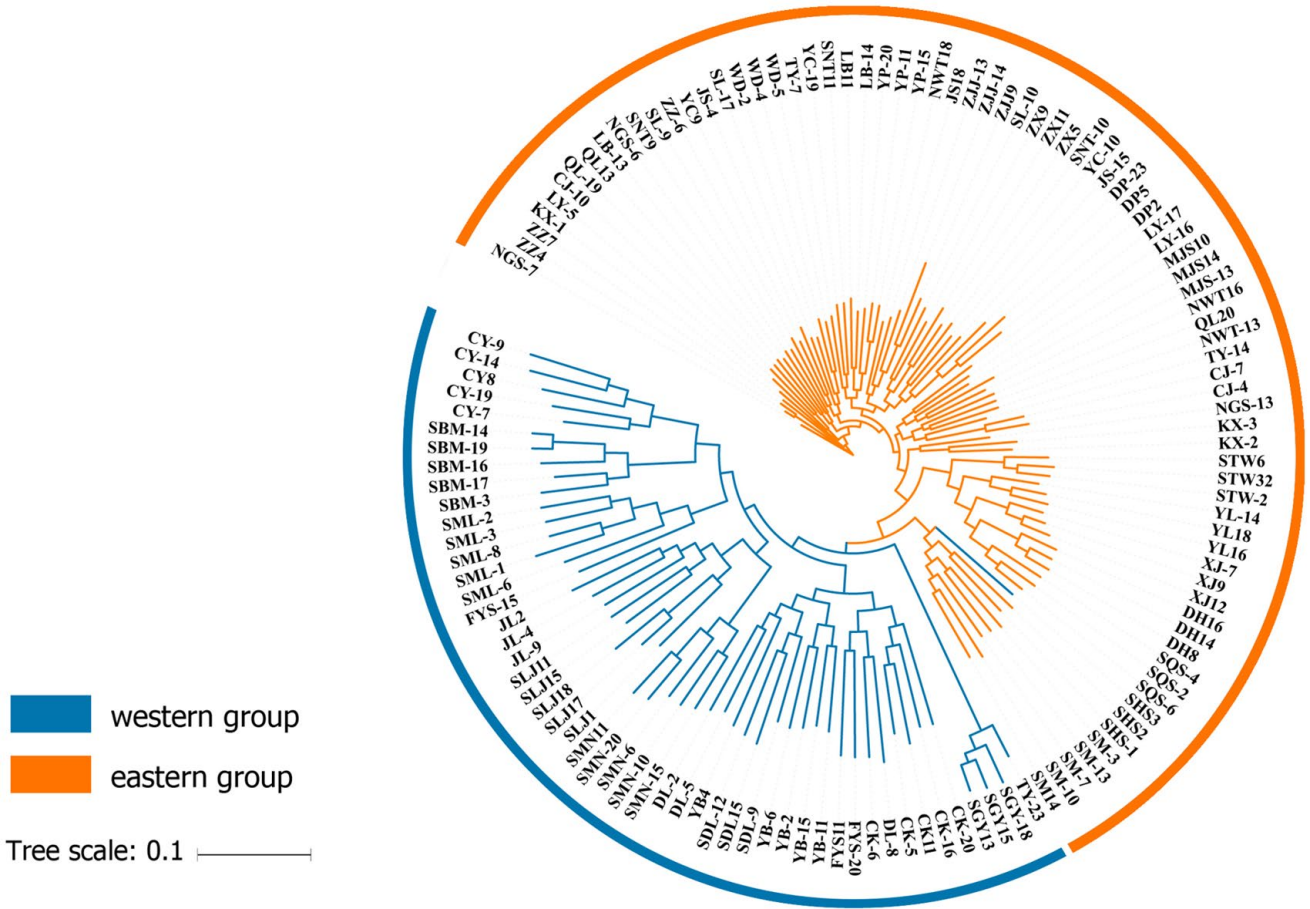
Dendrogram obtained for the *Q. spinosa* populations based on genetic distance.

**Figure 4:**
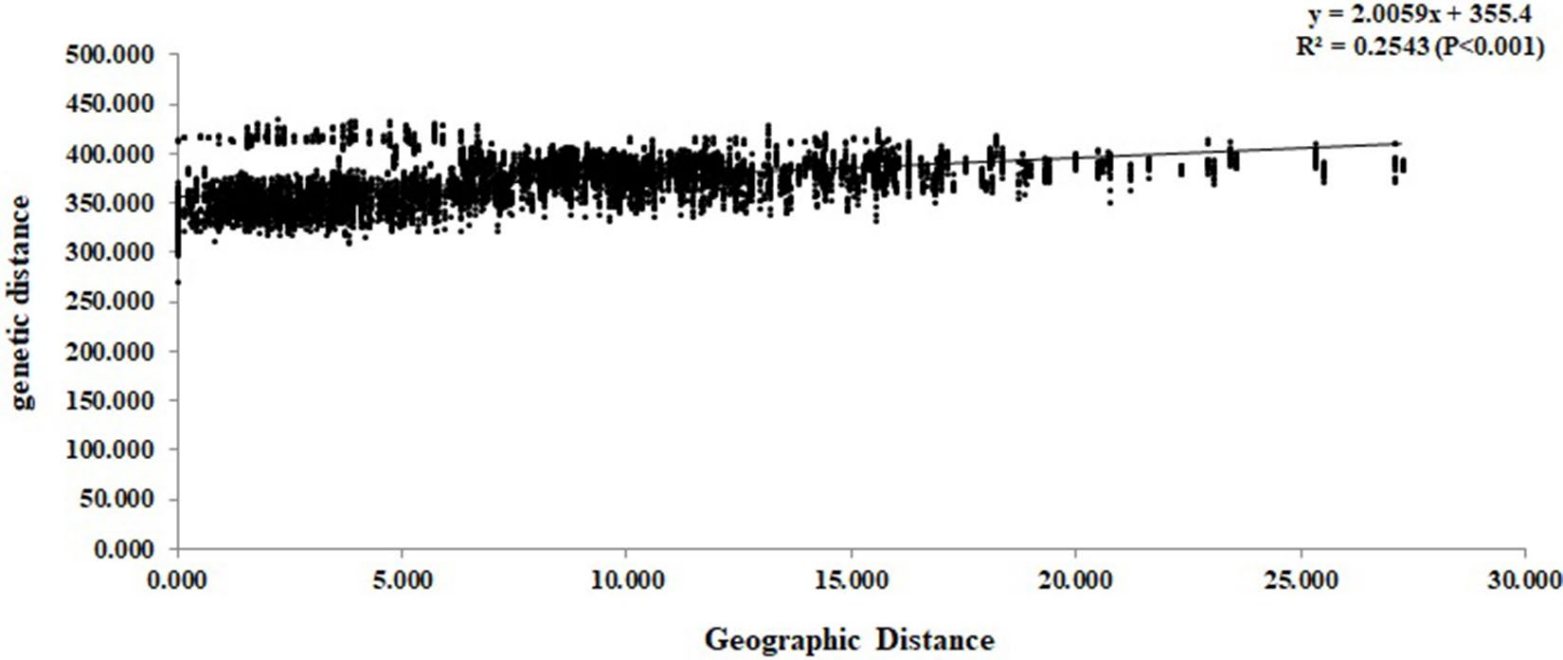
Mantel test between genetic distance and geographic distance among the *Q. spinosa* individuals.

At the individual level, the genetic relationship was consistent with the geographical gradient of genomic introgression. Individuals which were geographically proximal to genetic center contained more ancestry from the cluster. The dendrogram suggested that the clustering highly depended on the geographic origins of populations (**Figure 3**). The results demonstrated a closer relationship among individuals in the western group. These populations were mainly distributed in the HHM. In another cluster branch, the individuals were mainly from the eastern group whose populations located in Qinling Mountains, Central China, and Taiwan, China. Thus, the populations located on disparate mountain regions had distinct genetic characters. The results of the Mantel test also indicated that a positive relativity existed between the genetic distance and geographic distance regarding the examined individuals (R^2^ = 0.2543, p < 0.001, 999 permutations) (**Figure 4**).

### Demographic Modeling

**Figure 5:**
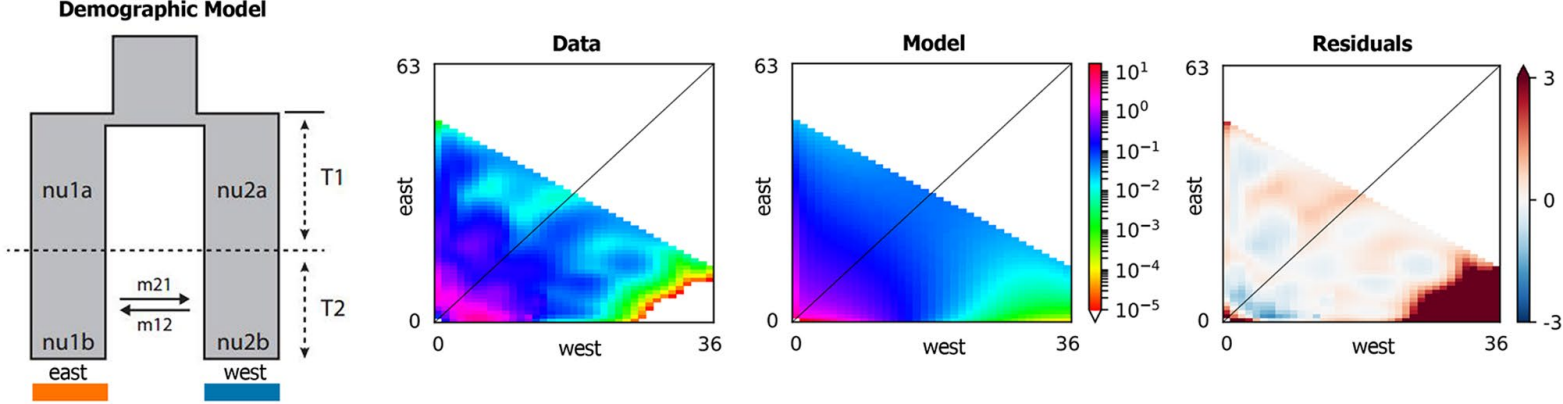
The best-supported model from δaδi analysis.

According to the result of ADMIXTURE, the populations of *Q. spinosa* were divided into two groups, that is, western group and eastern group. The site frequency spectrum (SFS) detection of individuals from each group was implemented first. Based on the maximum value of SFS and the number of individuals of each group, the final dataset was projected to a size of 52 individuals from western group and 78 individuals from eastern group (proj = [36, 63]), yielding 58,353 SNPs. Regarding all levels of complexity, the Sec_contact_asym_mig_size model with the lowest AIC scores (**Supplementary Table 3**) was favored over all other models. The best-supported demographic model had the features shown in **Figure 5**. In the highest-likelihood parameter set, the west and east groups within *Q. spinosa* had divergence in isolation for a long time (T1 = 8.15). Then the two groups in recent periods had the secondary contact with asymmetrical gene flow (T2 = 0.11). The direction of gene flow was from the western group to the eastern group during this period (m12 = 12.58 migrants/generation with a 95% CI: 11.00–20.47 vs. m21 = 1.46 with a 95% CI: 0.82–1.50).

### Ecological Niche Analyses

**Figure 6:**
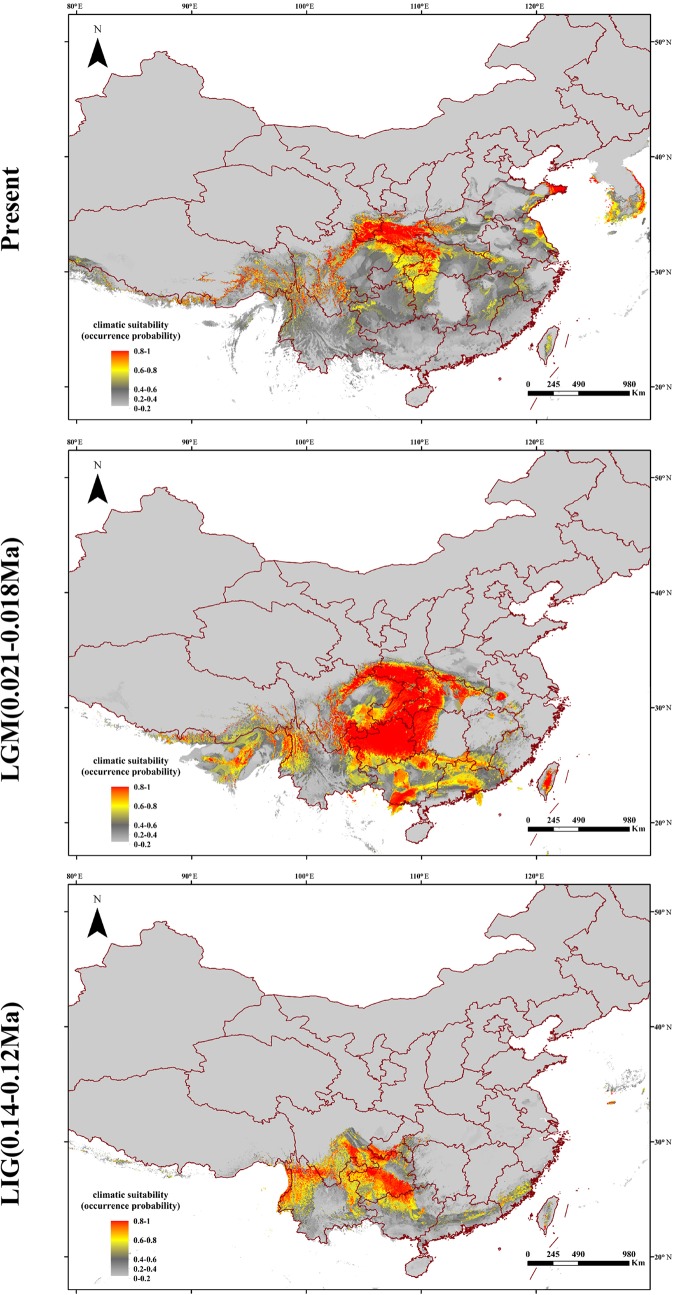
Predicted distributions of *Q. spinosa* based on ecological niche modeling.

Based on the collected location records of *Q. spinosa*, the geographic distribution maps were generated to predict areas where *Q. spinosa* might emerge. The model performance for *Q. spinosa* was better than random (AUC = 0.996). Thus, the model performed well in predicting the suitable habitat for the species. The ENMs revealed that the suitable areas for *Q. spinosa* in last interglacial period were mainly located in Hengduan Mountains. At the beginning of the glacial period, the temperature decreased. The suitable areas of *Q. spinosa* were expanded during the last glacial maximum. The species had the most extensive habitat in this period. At present, the climate became much warmer than the glacial period. The suitable habitat areas for *Q. spinosa* were predicted to locate in the Qin-ling Mountains and HHM. In the region located between the two mountains, the suitable habitats were continuous, which presented a strip-shaped distribution in the Sichuan Basin. In other regions, the ecological niche presented a fragmentation distribution (**Figure 6**).

## Discussions

### Population Structure and Divergence of *Q. spinosa*

To the best of our knowledge, the SNPs reported in this study were the most saturated markers yet obtained for *Q. spinosa*. The increased resolution supported by the genome-wide SNP data revealed that two clustering methods (i.e., -k-means clustering and the Bayesian model-based method) could get the consistency of individual membership assignment (**Figure 1**). Besides, the result of individual clustering was in concordance with the phylogenetic tree (**Figure 3**) and species geographical distribution pattern (**Figure 2**).

The patterns of population structure were divided into two groups. Group 1 consisted of individuals that belonged to the western regions, which were mainly distributed in the HHM. Group 2 consisted of individuals that were from the eastern regions, which were mainly distributed in the Qin-ling Mountains and Central China. The two groups had a relatively distinct genetic component, which may be a strong barrier for the gene flow between western lineages and eastern lineages. The phenomenon of two lineages was differences in the *Q. spinosa*, as indicated by previous studies in other species of subtropical China, such as *Ginkgo biloba* (Gong et al., 2008), *Quercus glauca* (Xu et al., 2015). These results supported the long-term geographic isolation and variation in ecological factors which catalyzed the high levels of intra-species genetic divergence.

*Q. spinosa* had the characteristics of long generation times, wind-pollinated, seed dispersal by gravity and rodent (Austerlitz et al., 2000). Most oak seeds were simply scattered around the mother tree by gravity, and only a few seeds crept down with the mountains topography or were carried limited distances by small rodents (e.g. squirrels and mice) and jays (Gómez, 2003; Gómez et al., 2003; Xiao et al., 2009). This physiological property resulted in high genetic differentiation intra-species.

The complex geological structure of HHM and the vertical shift of the climatic zone formed a natural geographical barrier and glacial refugia for alpine vegetation (Chen et al., 2011). The complex terrain of mountains acts as an effective geographical barrier for the oak seeding or pollen dispersal (Liu et al., 2013; Li et al., 2014), and has provided potential conditions for the independent evolution of the two lineages. The results of Mantel test showed that there was a significant positive correlation between the genetic distance and geographic distance of individuals (R^2^ = 0.2153, p < 0.001, 999 permutations) (**Figure 4**). The results also illustrated that the gullies of mountain potential and complex terrain slopes caused the existence of strong barriers for the gene exchange between two lineages. In fact, similar genetic structure was also detected in other species in this region, such as *Garrulax elliotii* from the eastern Himalays (Qu et al., 2011) and *Taxus wallichiana* from Himalaya-Hengduan Mountains region (Liu et al., 2013).

### Demographical History of *Q. spinosa*

The first uplift of Qinghai-Tibet Plateau (QTP) began around 40 Ma (Harrison et al., 1992). Some studies showed that the main uplifting of QTP occurred in 10 Ma (Royden et al., 2008), the plateau was uplifted above 4,000 m during 3.4 to 1.6 Ma, and reached peak elevation before the Late Pliocene (Li et al., 1996).

The hotspots of biodiversity are often associated with areas that have undergone orogenic activities during recent geological history. The recent dramatic uplift of QTP had significant effect on species differentiation and genetic structure (Li et al., 1996). Previous studies showed that the spatial distribution pattern of *Quercus sect. Heterobalanus* was closely related to the uplift of the HHM (Zhou et al., 2003; Feng et al., 2016). The early Pliocene accompanied by simultaneous cooling triggered the differentiation of oaks (Meng et al., 2017), and the mountains uplifts catalyzed the species radiation of sclerophyllous forests (Du et al., 2017).

The δaδi analyses (**Figure 5**) indicated that two lineages (west lineages and east lineages) might be diverged from the ancestral lineage for a long time, and the result indicated that divergence of the *Q. spinosa* might be caused by the recent rapid uplift of the QTP during Miocene (10–8 Ma) (Harrison et al., 1992; Royden et al., 2008; Searle, 2011). The enhanced monsoon climate triggered differentiation among two groups after the uplifting of the QTP at 15–13 Ma (An et al., 2001; Wan et al., 2007; Jacques et al., 2011). This might result in lots of small fragmented habitats with different microclimate, which could influence the direction of natural selection (Sobel et al., 2010), and be responsible for the high differentiation within species. The divergence times were significantly consistent with a period for the uplift of the QTP and the formation of HHM (Tapponnier et al., 1990; Shi et al., 1999), which indicated that the QTP uplifting during Pliocene might result in fragmented habitats with species, and promote the intra-species differentiation. The two lineages diverged during the Neogene and remained separated thereafter.

The Quaternary glacial turbulence had a significant effect on species distribution ranges (Comes and Kadereit, 1998; Hewitt, 2004). The effects of glacial expansions and contractions on genetic heritage largely depend on the latitude and topography in various parts of the globe. Most plant taxa were supposed to have the shift of latitude or elevation ranges in response to glaciation (Davis and Shaw, 2001). The theory was also applicable in *Q. spinosa*. During the glacial periods, the monsoons were blocked by the North-South ranges in the HHM regions (Liu et al., 2013). Therefore, the western populations merely migrated vertically to the high altitude. However, a universal phenomenon that plants in Asian continent migrated from north to south during the period of climate cooling. For example, *Larix gmelinii* migrated from southern Da Hinggan Mountains to the North China and formed a new varietas, *Larix gmelinii* var. *principis-ruprechtii* (Chen et al., 2011). The eastern populations of *Q. spinosa* were mainly distributed in mountains of West-East range, like Qin-ling Mountains or Central China. Hence, the migration of eastern populations likewise expanded to the south region during glacial periods. The ENM consistently indicated that the suitable habitat of *Q. spinosa* were continuous in the west regions and some fragmentations of the eastern regions (**Figure 6**). The ecological differences found from the studies of the two *Q. spinosa* lineages were sufficient for separating both lineages in a high degree, as indicated by the genetic data. Such ecological niche partitioning reinforced the divergence of the two lineages after the initial spatial isolation, and the species might have got some differential adaptation regarding the environmental conditions.

With the uplift of QTP, great changes took place in the HHM environment. The climate became cold and dry. Though the temperature on the QTP increased at the end of the largest glaciation (ca. 1.2–0.6 Ma), the cold climate might continue until the late Ionian stage (0.3–0.126 Ma) (Shi et al., 1999). However, sclerophyllous oaks could adapt to cold habitats due to inherent physiological characteristics (Zhou, 1993). It is feasible that *Q. spinosa* expanded their ranges and increased their population sizes during this period. The moderately cold climate prevailing in the HHM region provided opportunities for *Q. spinosa* to continue its ranges expansion, and gradually catalyzed the species to become the constructive and dominant species in the HHM (Zhou, 1992). Besides, the ENM analyses suggested that *Q. spinosa* had experienced ranges expansion from LIG (0.14–0.12 Ma) to LGM (0.021–0.018 Ma), which was similar to other alpine plants reported in this region, e.g., *Picea likiangensis*, *Picea wilsonii* (Li et al., 2014; Sun et al., 2015), and *Taxus wallichiana* (Liu et al., 2013). The demographic modeling verified the conclusion, and also explained that the two lineages had gene exchange at Sichuan Basin. The Sichuan Basin was a region of transitional flora for the evergreen broadleaf forest, especially for Fagaceae (He et al., 2005). The suitable modeling showed that the two groups experienced the secondary contact at LGM (0.11 Ma), and the asymmetrical gene flowed from the west region to the east region (m12 = 12.58 vs. m21 = 1.46). Therefore, climatic oscillations during the Pleistocene had pronounced effects on the recent population history of *Q. spinosa*. Two lineages evolved in allopatry through climate/orogeny-induced vicariance, and had the secondary contact after last glacial maximum. This genetic differentiation model for *Q. spinosa* was similar to the other Fagaceae species (e.g., *Castanopsis carlesii*) (Sun et al., 2016). The genetic data and ecological niche reconstruction consistently identified that two lineages mostly maintained by populations’ environmental adaptation.

## Conclusions

This study used thousands of genome-wide SNPs to produce the first range-wide map of demographic model in *Q. spinosa*. All reads passed the quality controls. Finally, 58,353 consensus SNPs were developed for the population genetics analyses. The results indicated that large amounts of high quality reads almost covered the entire genome, which provided more comprehensive theoretical basis for the following research. The multidisciplinary approach combining molecular markers, ENMs, and demographic modeling strongly supported that the geological and climatic event since Miocene, and Quaternary climatic oscillations triggered the differentiation, evolution, and range shifts of *Q. spinosa*. The results promote deep insights into the diversification and evolutionary dynamics of sclerophyllous species in the HHM.

## Data Availability Statement

Publicly available datasets were analyzed in this study. These data can be found here: http://www.ncbi.nlm.nih.gov/bioproject/549662. 

## Author Contributions

G-FZ and M-MJ contributed to the conception of the study. LF and JY collected the materials. M-MJ performed the data analyses and wrote the manuscript. LF, JY, Y-CY, and X-DC helped perform the analysis with constructive discussions. All authors contributed critically to the drafts and gave final approval for publication.

## Funding

This work was financially supported by the National Natural Science Foundation of China (31901075, 31770229).

## Conflict of Interest

The authors declare that the research was conducted in the absence of any commercial or financial relationships that could be construed as a potential conflict of interest.

## Supplementary Material

Figure S1 Geographic origin of the Q.spinosa populations sampled in the study.

Supplementary Table 1 Specimen and sample information for SLAF-seq analyses.

Supplementary Table 2 The statistics of the Q.spinosa initial sequencing data statistics describing the distribution of different properties of each sequenced individual.

Supplementary Table 3 Summary of the likelihoods for the 32 demographic models tested.
