# Supplementary figures and images for: Evaluating Population Genetic Structure and Demographic History of Quercus spinosa (Fagaceae) Based on Specific Length Amplified Fragment Sequencing
Source: Front Genet. 2019 Oct 3;10:965. doi: 10.3389/fgene.2019.00965 (PMC6785805; doi:10.3389/fgene.2019.00965)

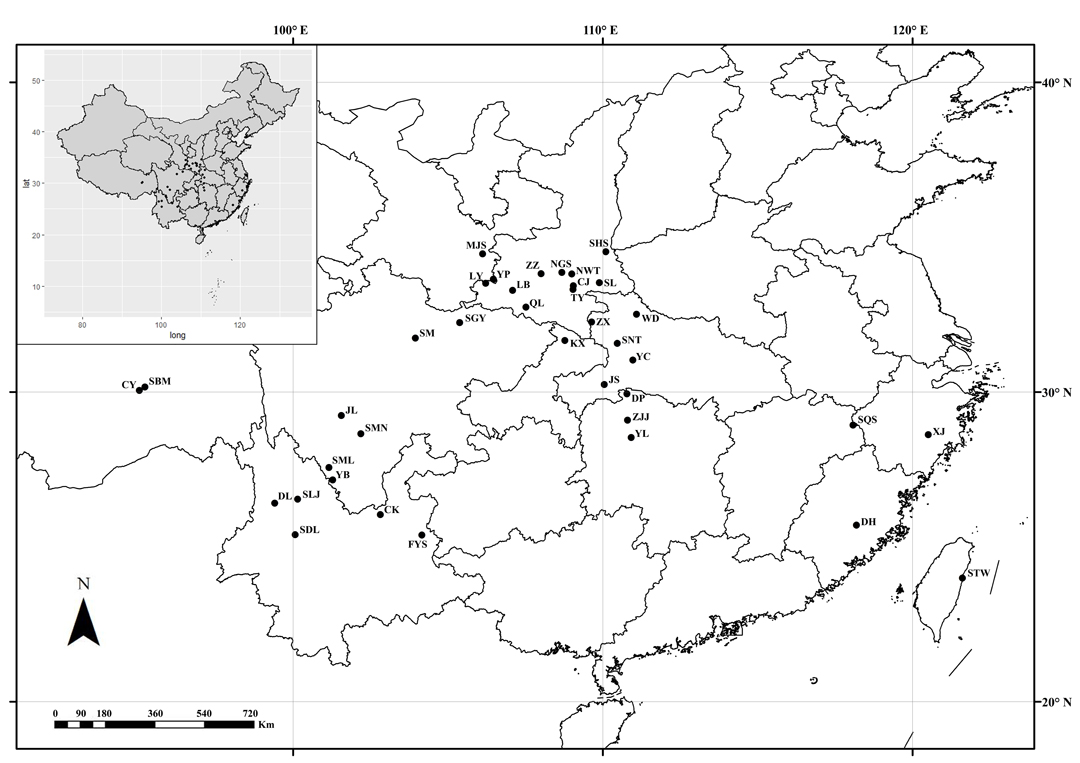

Supplement: Figure S1 — Geographic origin of the Q.spinosa populations sampled in the study. [file Image_1.jpeg]
