# Supplementary material for: Evaluating Population Genetic Structure and Demographic History of Quercus spinosa (Fagaceae) Based on Specific Length Amplified Fragment Sequencing
Source: Front Genet. 2019 Oct 3;10:965. doi: 10.3389/fgene.2019.00965 (PMC6785805; doi:10.3389/fgene.2019.00965)
Supplement: Supplementary Table 1 — Specimen and sample information for SLAF-seq analyses. [file Table_1.docx]

Supplementary Table 1: Specimen and sample information for SLAF-seq analyses

| ID | N | longitude | Latitude |
| --- | --- | --- | --- |
| QL | 3 | 107.52 | 32.74 |
| SGY | 3 | 105.38 | 32.24 |
| SBM | 5 | 95.22 | 30.16 |
| CY | 5 | 95.04 | 30.05 |
| SML | 5 | 101.16 | 27.56 |
| SLJ | 5 | 100.15 | 26.54 |
| LB | 3 | 107.09 | 33.28 |
| XJ | 3 | 120.51 | 28.62 |
| SQS | 3 | 118.08 | 28.93 |
| JS | 3 | 110.05 | 30.24 |
| ZX | 3 | 109.64 | 32.26 |
| SNT | 3 | 110.47 | 31.57 |
| YC | 3 | 110.97 | 31.03 |
| ZZ | 3 | 108.01 | 33.82 |
| CJ | 3 | 109 | 33.81 |
| NGS | 3 | 109.05 | 33.43 |
| ZJJ | 3 | 110.8 | 29.09 |
| YL | 3 | 110.92 | 28.53 |
| SDL | 3 | 100.07 | 25.39 |
| MJS | 3 | 106.12 | 34.46 |
| NWT | 3 | 108.68 | 33.86 |
| SL | 3 | 109.89 | 33.53 |
| LY | 3 | 106.22 | 33.51 |
| SHS | 3 | 110.1 | 34.53 |
| DH | 3 | 118.19 | 25.7 |
| YB | 5 | 101.28 | 27.16 |
| SM | 5 | 103.95 | 31.74 |
| SMN | 5 | 102.19 | 28.65 |
| KX | 3 | 108.7778 | 31.6632 |
| STW | 3 | 121.61 | 23.99 |
| FYS | 3 | 104.16 | 25.38 |
| CK | 5 | 102.82 | 26.04 |
| DP | 3 | 110.78 | 29.94 |
| JL | 3 | 101.56 | 29.24 |
| YP | 3 | 106.46766 | 33.641495 |
| WD | 3 | 111.09 | 32.51 |
| TY | 3 | 109.04 | 33.31 |
| DL | 3 | 99.41 | 26.41 |
