# Supplementary material for: Evaluating Population Genetic Structure and Demographic History of Quercus spinosa (Fagaceae) Based on Specific Length Amplified Fragment Sequencing
Source: Front Genet. 2019 Oct 3;10:965. doi: 10.3389/fgene.2019.00965 (PMC6785805; doi:10.3389/fgene.2019.00965)
Supplement: Supplementary Table 2 — The statistics of the Q.spinosa initial sequencing data statistics describing the distribution of different properties of each sequenced individual. [file Table_2.docx]

Supplementary Table 2: The statistics of the *Q.spinosa* initial sequencing data

Statistics describing the distribution of different properties of each sequenced individual

| Sample ID | Total Reads | Q30 Percentage(%) | GC Percentage(%) | SLAF number | Total depth | Average depth | Total SNP | SNP num | Integrity | Heter ratio |
| --- | --- | --- | --- | --- | --- | --- | --- | --- | --- | --- |
| QL13 | 1509954 | 89.73 | 41.39 | 129,928 | 998,769 | 7.69 | 124,607 | 81,093 | 64.93% | 8.48% |
| QL20 | 1892445 | 90 | 40.63 | 152,858 | 1,243,835 | 8.14 | 124,607 | 88,752 | 71.07% | 9.74% |
| QL-19 | 1887701 | 93.99 | 41.92 | 173,235 | 1,271,346 | 7.34 | 124,607 | 87,730 | 70.25% | 10.38% |
| SGY13 | 2248577 | 93.86 | 40.3 | 138,326 | 1,415,905 | 10.24 | 124,607 | 61,379 | 49.15% | 6.20% |
| SGY-18 | 2089685 | 94.05 | 40.77 | 147,002 | 1,269,201 | 8.63 | 124,607 | 62,154 | 49.77% | 7.20% |
| SGY15 | 2330250 | 87.83 | 38.89 | 110,903 | 1,321,248 | 11.91 | 124,607 | 52,595 | 42.11% | 4.72% |
| SBM-14 | 1682579 | 93.35 | 41.42 | 149430 | 1115275 | 7.46 | 124607 | 85018 | 0.6808 | 0.1047 |
| SBM-16 | 1940579 | 91.92 | 38.15 | 140002 | 1185230 | 8.47 | 124607 | 68345 | 0.5473 | 0.0999 |
| SBM-17 | 2046378 | 93.33 | 40.91 | 166716 | 1345375 | 8.07 | 124607 | 85743 | 0.6866 | 0.117 |
| SBM-19 | 1888648 | 92.82 | 41.74 | 164875 | 1209139 | 7.33 | 124607 | 89928 | 0.7201 | 0.1329 |
| SBM-3 | 1600784 | 92.64 | 41.85 | 148961 | 1024342 | 6.88 | 124607 | 84573 | 0.6772 | 0.1111 |
| CY8 | 2974467 | 90.18 | 40.59 | 132289 | 2002379 | 15.14 | 124607 | 92210 | 0.7384 | 0.1356 |
| CY-14 | 2993864 | 93.67 | 41.29 | 175,008 | 1,839,240 | 10.51 | 124,607 | 99,255 | 79.48% | 14.61% |
| CY-19 | 2278921 | 93 | 41.95 | 125,729 | 1,421,197 | 11.3 | 124,607 | 87,679 | 70.21% | 11.05% |
| CY-7 | 3421710 | 93.07 | 42.9 | 160,830 | 2,197,920 | 13.67 | 124,607 | 92,334 | 73.94% | 14.04% |
| CY-9 | 2361325 | 92.97 | 40.98 | 139,723 | 1,467,959 | 10.51 | 124,607 | 93,181 | 74.61% | 12.58% |
| SML-1 | 2762815 | 93.4 | 39.8 | 161,830 | 1,847,872 | 11.42 | 124,607 | 93,218 | 74.64% | 14.90% |
| SML-2 | 3166082 | 93.71 | 41.01 | 171,593 | 2,156,922 | 12.57 | 124,607 | 101,106 | 80.96% | 15.01% |
| SML-3 | 2058048 | 93.43 | 40.5 | 145,273 | 1,415,403 | 9.74 | 124,607 | 93,457 | 74.83% | 13.63% |
| SML-6 | 1962754 | 92.94 | 41.24 | 134,691 | 1,324,608 | 9.83 | 124,607 | 84,935 | 68.01% | 10.64% |
| SML-8 | 6844685 | 93.19 | 38.93 | 212,997 | 4,745,397 | 22.28 | 124,607 | 100,738 | 80.67% | 16.48% |
| SLJ17 | 2738167 | 88.91 | 40.48 | 152,809 | 1,733,583 | 11.34 | 124,607 | 92,155 | 73.79% | 11.88% |
| SLJ18 | 1964352 | 89.35 | 41.39 | 133,286 | 1,266,507 | 9.5 | 124,607 | 85,265 | 68.27% | 11.60% |
| SLJ1 | 2413623 | 88.72 | 40.34 | 134,211 | 1,517,671 | 11.31 | 124,607 | 82,594 | 66.14% | 10.96% |
| SLJ11 | 2169656 | 88.72 | 40.64 | 128,119 | 1,334,506 | 10.42 | 124,607 | 81,479 | 65.24% | 10.67% |
| SLJ15 | 2966399 | 89.54 | 40.91 | 150,854 | 1,879,339 | 12.46 | 124,607 | 87,757 | 70.27% | 11.73% |
| LB-13 | 2213088 | 93.75 | 40.22 | 158,998 | 1,514,943 | 9.53 | 124,607 | 87,603 | 70.15% | 9.88% |
| LB-14 | 2358356 | 93.45 | 41.09 | 157,203 | 1,615,718 | 10.28 | 124,607 | 88,529 | 70.89% | 10.41% |
| LB11 | 2132272 | 89.87 | 39.82 | 124,952 | 1,415,464 | 11.33 | 124,607 | 89,270 | 71.48% | 10.45% |
| XJ9 | 3583823 | 89.77 | 40.3 | 154,362 | 2,308,898 | 14.96 | 124,607 | 90,787 | 72.70% | 7.51% |
| XJ12 | 2456757 | 89.72 | 39.35 | 128,109 | 1,601,871 | 12.5 | 124,607 | 85,672 | 68.60% | 8.38% |
| XJ-7 | 1816165 | 93.07 | 39.59 | 140,338 | 1,180,850 | 8.41 | 124,607 | 83,606 | 66.95% | 7.54% |
| SQS-2 | 2601129 | 93.34 | 39.18 | 151,820 | 1,732,692 | 11.41 | 124,607 | 82,041 | 65.69% | 7.43% |
| SQS-4 | 2604587 | 93.07 | 39.73 | 172,811 | 1,514,223 | 8.76 | 124,607 | 92,433 | 74.01% | 9.74% |
| SQS-6 | 2321649 | 93.01 | 40.33 | 146,588 | 1,543,511 | 10.53 | 124,607 | 85,964 | 68.83% | 7.57% |
| JS18 | 2449911 | 89.07 | 39.96 | 131,638 | 1,571,182 | 11.94 | 124,607 | 86,540 | 69.30% | 9.27% |
| JS-15 | 2957180 | 93.61 | 40.45 | 147,458 | 1,775,978 | 12.04 | 124,607 | 98,178 | 78.62% | 12.26% |
| JS-4 | 1854213 | 93.62 | 41.08 | 162,565 | 1,251,931 | 7.7 | 124,607 | 87,965 | 70.44% | 9.99% |
| ZX5 | 2148464 | 90.1 | 39.91 | 132,546 | 1,409,315 | 10.63 | 124,607 | 83,218 | 66.64% | 9.45% |
| ZX9 | 2583901 | 89.58 | 39.74 | 134,352 | 1,662,350 | 12.37 | 124,607 | 91,443 | 73.22% | 10.99% |
| ZX11 | 2239597 | 88.96 | 39.47 | 130,033 | 1,401,085 | 10.77 | 124,607 | 87,134 | 69.77% | 11.41% |
| SNT-10 | 2010974 | 91.33 | 39.2 | 126,376 | 1,263,852 | 10 | 124,607 | 85,620 | 68.56% | 10.78% |
| SNT11 | 1895498 | 89.64 | 41.75 | 143,209 | 1,190,995 | 8.32 | 124,607 | 85,030 | 68.09% | 8.94% |
| SNT9 | 1522653 | 90.26 | 41.34 | 128,966 | 1,007,143 | 7.81 | 124,607 | 80,252 | 64.26% | 8.83% |
| YC9 | 2767271 | 90.35 | 41.76 | 180,699 | 1,798,672 | 9.95 | 124,607 | 91,037 | 72.90% | 10.39% |
| YC-10 | 2856865 | 90.91 | 40 | 143,442 | 1,757,867 | 12.25 | 124,607 | 92,501 | 74.07% | 11.69% |
| YC-19 | 1737487 | 93.25 | 39.81 | 136,968 | 1,166,986 | 8.52 | 124,607 | 84,852 | 67.94% | 9.26% |
| ZZ-6 | 1730264 | 91.99 | 40.71 | 134,430 | 1,096,808 | 8.16 | 124,607 | 84,360 | 67.55% | 9.52% |
| ZZ7 | 2345921 | 88.6 | 39.86 | 130,718 | 1,479,131 | 11.32 | 124,607 | 92,829 | 74.33% | 11.70% |
| ZZ4 | 2743999 | 89.04 | 39.54 | 137,963 | 1,763,422 | 12.78 | 124,607 | 88,359 | 70.75% | 9.78% |
| CJ-10 | 3086668 | 93.22 | 41.11 | 158,016 | 1,989,472 | 12.59 | 124,607 | 94,723 | 75.85% | 11.05% |
| CJ-4 | 2334884 | 93.13 | 41.01 | 146,107 | 1,559,034 | 10.67 | 124,607 | 89,909 | 71.99% | 10.03% |
| CJ-7 | 2721075 | 92.8 | 40.57 | 132,795 | 1,660,590 | 12.5 | 124,607 | 92,060 | 73.72% | 10.44% |
| NGS-13 | 2440716 | 93.48 | 41.35 | 177,863 | 1,660,199 | 9.33 | 124,607 | 92,095 | 73.74% | 10.50% |
| NGS-6 | 2379433 | 92.6 | 40.19 | 154,471 | 1,574,144 | 10.19 | 124,607 | 87,272 | 69.88% | 10.12% |
| NGS-7 | 2522516 | 93.77 | 39.71 | 156,105 | 1,743,610 | 11.17 | 124,607 | 87,889 | 70.38% | 9.83% |
| ZJJ-13 | 2358660 | 90.71 | 40.19 | 142,418 | 1,385,785 | 9.73 | 124,607 | 86,118 | 68.96% | 10.00% |
| ZJJ9 | 2469422 | 89.58 | 40.56 | 134,856 | 1,616,776 | 11.99 | 124,607 | 84,753 | 67.86% | 8.35% |
| ZJJ-14 | 1581634 | 88.14 | 40.11 | 108,355 | 966,743 | 8.92 | 124,607 | 79,336 | 63.53% | 8.70% |
| YL16 | 2439856 | 90.03 | 42.03 | 147,933 | 1,565,877 | 10.59 | 124,607 | 82,396 | 65.98% | 6.95% |
| YL18 | 2958517 | 90.16 | 41.83 | 158,242 | 1,924,376 | 12.16 | 124,607 | 86,198 | 69.02% | 8.20% |
| YL-14 | 1951410 | 92.92 | 39.96 | 135,835 | 1,266,673 | 9.33 | 124,607 | 77,414 | 61.99% | 7.98% |
| SDL-12 | 2212863 | 93.06 | 41.99 | 160,993 | 1,411,729 | 8.77 | 124,607 | 88,138 | 70.58% | 12.34% |
| SDL-9 | 2147737 | 92.42 | 40.96 | 168,951 | 1,318,503 | 7.8 | 124,607 | 89,318 | 71.52% | 13.40% |
| SDL15 | 1835131 | 89.38 | 41.16 | 128,913 | 1,182,398 | 9.17 | 124,607 | 80,710 | 64.63% | 10.99% |
| MJS10 | 4884615 | 89.77 | 39.22 | 166,372 | 3,239,435 | 19.47 | 124,607 | 94,593 | 75.74% | 11.08% |
| MJS14 | 1335439 | 89.64 | 40.37 | 104,504 | 866,247 | 8.29 | 124,607 | 73,652 | 58.98% | 8.34% |
| MJS-13 | 1923512 | 93.06 | 41.57 | 151,185 | 1,277,556 | 8.45 | 124,607 | 84,454 | 67.63% | 10.20% |
| NWT16 | 1806500 | 90.23 | 42.18 | 153,654 | 1,180,703 | 7.68 | 124,607 | 82,759 | 66.27% | 9.16% |
| NWT18 | 2065720 | 90.58 | 41.32 | 152,002 | 1,378,412 | 9.07 | 124,607 | 88,892 | 71.18% | 9.88% |
| NWT-13 | 1757770 | 92.94 | 39.92 | 153,417 | 1,119,552 | 7.3 | 124,607 | 78,508 | 62.86% | 9.38% |
| SL-10 | 1935330 | 93.51 | 41.03 | 161,295 | 1,162,671 | 7.21 | 124,607 | 95,176 | 76.21% | 12.65% |
| SL-9 | 1767185 | 93.46 | 41.97 | 159,514 | 1,125,709 | 7.06 | 124,607 | 84,824 | 67.92% | 9.99% |
| SL-17 | 2332286 | 92.93 | 39.95 | 152,552 | 1,547,500 | 10.14 | 124,607 | 88,961 | 71.23% | 10.09% |
| LY-16 | 2207350 | 93.45 | 41.5 | 160,495 | 1,496,240 | 9.32 | 124,607 | 89,096 | 71.34% | 10.82% |
| LY-17 | 1878433 | 93.48 | 42.17 | 159,768 | 1,217,382 | 7.62 | 124,607 | 82,476 | 66.04% | 10.54% |
| LY-5 | 1886654 | 93.62 | 40.69 | 148,430 | 1,253,112 | 8.44 | 124,607 | 83,586 | 66.93% | 9.41% |
| SHS-1 | 2184108 | 92.35 | 41.28 | 170,113 | 1,337,907 | 7.86 | 124,607 | 92,483 | 74.05% | 13.24% |
| SHS2 | 1888460 | 89.58 | 41.59 | 133,407 | 1,216,184 | 9.12 | 124,607 | 83,114 | 66.55% | 10.46% |
| SHS3 | 2712376 | 89.97 | 40.91 | 152,241 | 1,769,869 | 11.63 | 124,607 | 90,953 | 72.83% | 12.68% |
| DH16 | 1125840 | 88.51 | 39.61 | 115,849 | 626,618 | 5.41 | 124,607 | 71,048 | 56.89% | 7.76% |
| DH8 | 1798961 | 89.72 | 41.43 | 108,796 | 1,181,444 | 10.86 | 124,607 | 69,586 | 55.72% | 6.08% |
| DH14 | 2345063 | 88.35 | 40.19 | 133,223 | 1,395,104 | 10.47 | 124,607 | 81,064 | 64.91% | 6.67% |
| YB4 | 1731734 | 90.42 | 41.47 | 134,875 | 1,132,513 | 8.4 | 124,607 | 83,414 | 66.79% | 11.06% |
| YB-11 | 2520771 | 93.31 | 40.94 | 160,635 | 1,662,547 | 10.35 | 124,607 | 92,510 | 74.08% | 13.19% |
| YB-15 | 2392955 | 92.91 | 39.87 | 157,849 | 1,549,860 | 9.82 | 124,607 | 90,111 | 72.16% | 12.93% |
| YB-2 | 2336871 | 93.4 | 41.97 | 160,574 | 1,527,346 | 9.51 | 124,607 | 92,198 | 73.83% | 13.46% |
| YB-6 | 2659513 | 92.72 | 40.78 | 165,392 | 1,728,592 | 10.45 | 124,607 | 93,457 | 74.83% | 13.65% |
| SM-10 | 1868924 | 93.34 | 40.85 | 135,465 | 1,247,412 | 9.21 | 124,607 | 83,472 | 66.84% | 10.28% |
| SM-13 | 2706909 | 93.06 | 40.8 | 158,586 | 1,784,585 | 11.25 | 124,607 | 92,305 | 73.91% | 12.51% |
| SM14 | 2807301 | 93.06 | 40.47 | 155,269 | 1,886,406 | 12.15 | 124,607 | 89,742 | 71.86% | 11.64% |
| SM-3 | 2448625 | 93.83 | 40.85 | 165,000 | 1,636,365 | 9.92 | 124,607 | 88,066 | 70.52% | 11.46% |
| SM-7 | 3810484 | 93.03 | 40.33 | 182,469 | 2,585,243 | 14.17 | 124,607 | 95,481 | 76.46% | 12.63% |
| SMN-10 | 2165591 | 92.1 | 40.24 | 138,534 | 1,329,182 | 9.59 | 124,607 | 82,905 | 66.38% | 10.81% |
| SMN-15 | 2456673 | 91.56 | 39.8 | 140,345 | 1,506,120 | 10.73 | 124,607 | 85,074 | 68.12% | 10.54% |
| SMN-20 | 1752473 | 93.09 | 40.14 | 130,970 | 1,134,148 | 8.66 | 124,607 | 79,083 | 63.32% | 9.91% |
| SMN-6 | 3459758 | 93.47 | 40.46 | 169,715 | 2,285,997 | 13.47 | 124,607 | 92,810 | 74.32% | 10.74% |
| SMN11 | 1738677 | 88.94 | 41.79 | 121,141 | 1,082,524 | 8.94 | 124,607 | 76,459 | 61.22% | 9.27% |
| KX-1 | 2293852 | 93.57 | 41.45 | 163,544 | 1,542,956 | 9.43 | 124,607 | 88,328 | 70.73% | 10.17% |
| KX-2 | 2243753 | 93.25 | 41.39 | 157,641 | 1,464,972 | 9.29 | 124,607 | 89,459 | 71.63% | 12.07% |
| KX-3 | 1882483 | 93.54 | 41.21 | 164,434 | 1,097,312 | 6.67 | 124,607 | 85,491 | 68.46% | 11.56% |
| STW-2 | 3410534 | 93.91 | 39.24 | 197,803 | 2,256,146 | 11.41 | 124,607 | 82,292 | 65.89% | 9.00% |
| STW32 | 1630031 | 89.36 | 41.03 | 122,556 | 1,012,451 | 8.26 | 124,607 | 78,076 | 62.52% | 6.94% |
| STW6 | 1683557 | 90.16 | 41.58 | 133,303 | 1,048,042 | 7.86 | 124,607 | 78,689 | 63.01% | 7.10% |
| FYS-15 | 1836037 | 92.99 | 41.38 | 132,488 | 1,128,438 | 8.52 | 124,607 | 92,493 | 74.06% | 14.12% |
| FYS-20 | 2682653 | 93.1 | 40.86 | 160,620 | 1,641,197 | 10.22 | 124,607 | 97,586 | 78.14% | 14.04% |
| FYS11 | 2170421 | 90.19 | 40.38 | 121,079 | 1,448,606 | 11.96 | 124,607 | 85,771 | 68.68% | 11.99% |
| CK-16 | 2987578 | 93.24 | 40.1 | 164,512 | 1,868,624 | 11.36 | 124,607 | 92,872 | 74.37% | 13.19% |
| CK-20 | 3660111 | 93.1 | 41 | 157,654 | 2,466,505 | 15.65 | 124,607 | 96,173 | 77.01% | 13.93% |
| CK-5 | 3392458 | 93.35 | 41.49 | 156,130 | 2,254,169 | 14.44 | 124,607 | 95,303 | 76.31% | 13.33% |
| CK-6 | 3124035 | 93.07 | 42.61 | 142,537 | 1,894,531 | 13.29 | 124,607 | 98,845 | 79.15% | 16.41% |
| CK11 | 3378500 | 89.83 | 39.69 | 132,224 | 2,250,356 | 17.02 | 124,607 | 88,875 | 71.17% | 12.46% |
| DP2 | 1673015 | 88.41 | 40.07 | 129,084 | 1,012,302 | 7.84 | 124,607 | 91,057 | 72.91% | 11.05% |
| DP5 | 1480873 | 88.85 | 39.85 | 104,457 | 933,981 | 8.94 | 124,607 | 79,494 | 63.65% | 9.63% |
| DP-23 | 2068760 | 93.25 | 41.1 | 147,360 | 1,212,967 | 8.23 | 124,607 | 96,692 | 77.43% | 13.16% |
| JL-4 | 2190596 | 92.41 | 40.14 | 124,487 | 1,379,802 | 11.08 | 124,607 | 85,662 | 68.59% | 10.70% |
| JL-9 | 2309913 | 92.43 | 40.45 | 121,370 | 1,322,792 | 10.9 | 124,607 | 92,660 | 74.20% | 12.25% |
| JL2 | 1598796 | 89.41 | 39.95 | 105,927 | 1,009,817 | 9.53 | 124,607 | 82,356 | 65.95% | 12.20% |
| YP-11 | 2348315 | 92.86 | 40.05 | 169,574 | 1,477,574 | 8.71 | 124,607 | 90,155 | 72.19% | 10.64% |
| YP-15 | 2779407 | 92.89 | 41.33 | 165,408 | 1,846,862 | 11.17 | 124,607 | 93,674 | 75.01% | 11.18% |
| YP-20 | 2092280 | 93.09 | 41.13 | 153,938 | 1,393,791 | 9.05 | 124,607 | 90,555 | 72.51% | 10.46% |
| WD-2 | 2640805 | 93.13 | 40.01 | 166,346 | 1,768,802 | 10.63 | 124,607 | 93,277 | 74.69% | 10.97% |
| WD-4 | 2288701 | 92.77 | 40.27 | 158,446 | 1,495,521 | 9.44 | 124,607 | 90,754 | 72.67% | 9.79% |
| WD-5 | 2410478 | 92.5 | 40.7 | 141,089 | 1,604,235 | 11.37 | 124,607 | 88,145 | 70.58% | 10.06% |
| TY-14 | 2168817 | 92.34 | 41.84 | 167,388 | 1,365,048 | 8.16 | 124,607 | 92,364 | 73.96% | 11.38% |
| TY-23 | 2095739 | 91.87 | 39.76 | 130,203 | 1,193,826 | 9.17 | 124,607 | 61,332 | 49.11% | 6.20% |
| TY-7 | 2011255 | 92.81 | 40.88 | 143,044 | 1,336,224 | 9.34 | 124,607 | 83,949 | 67.22% | 9.11% |
| DL-2 | 2389707 | 93.34 | 41.84 | 129,207 | 1,434,875 | 11.11 | 124,607 | 90,150 | 72.19% | 12.46% |
| DL-5 | 2624740 | 92.81 | 41.05 | 140,992 | 1,666,376 | 11.82 | 124,607 | 92,651 | 74.19% | 12.33% |
| DL-8 | 3356774 | 93.23 | 40.11 | 160,242 | 2,020,510 | 12.61 | 124,607 | 101,150 | 81.00% | 15.78% |
