# Supplementary material for: Evaluating Population Genetic Structure and Demographic History of Quercus spinosa (Fagaceae) Based on Specific Length Amplified Fragment Sequencing
Source: Front Genet. 2019 Oct 3;10:965. doi: 10.3389/fgene.2019.00965 (PMC6785805; doi:10.3389/fgene.2019.00965)
Supplement: Supplementary Table 3 — Summary of the likelihoods for the 32 demographic models tested. [file Table_3.docx]

Supplementary Table 3: Summary of the likelihoods for the 32 demographic models tested

| Model | log-likelihood | AIC | chi-squared | theta | nu1a | nu2a | nu1b | nu2b | m12 | m21 | T1 | T2 | T3 |
| --- | --- | --- | --- | --- | --- | --- | --- | --- | --- | --- | --- | --- | --- |
| sec_contact_asym_mig_size | -399.11 | 814.22 | 120.81 | 7.69 | 2.7725 | 22.806 | 0.1392 | 0.4918 | 12.5837 | 1.4581 | 8.1464 | 0.1058 | - |
| sec_contact_sym_mig_size | -425.01 | 864.02 | 203.1 | 12.99 | 29.5567 | 0.7801 | 0.322 | 0.5306 | 4.947 | 6.1915 | 0.1918 | - | - |
| sec_contact_sym_mig_size_three_epoch | -426.98 | 869.96 | 201.01 | 12.13 | 0.4961 | 20.6839 | 0.5996 | 0.5398 | 4.8382 | 5.28 | 0.1719 | 0.0102 | - |
| sym_mig | -433.64 | 875.28 | 221.97 | 64.75 | 0.0962 | 0.127 | 13.128 | 0.0271 | - | - | - | - | - |
| sym_mig_size | -441.54 | 897.08 | 229.74 | 50.93 | 1.4944 | 0.7081 | 0.1464 | 0.2925 | 8.1846 | 0.8746 | 0.1117 | - | - |
| no_mig | -454.79 | 915.58 | 321.52 | 57.66 | 0.078 | 0.1124 | 0.0103 | - | - | - | - | - | - |
| sec_contact_sym_mig | -454.73 | 919.46 | 287.17 | 42.93 | 0.1086 | 0.1679 | 12.4869 | 19.0121 | 0.5859 | - | - | - | - |
| anc_sym_mig_size | -457.77 | 929.54 | 322.32 | 3.89 | 19.6578 | 6.0041 | 1.3889 | 1.4253 | 5.4238 | 26.5997 | 0.1557 | - | - |
| asym_mig | -462.82 | 935.64 | 259.09 | 44.77 | 0.1395 | 0.9548 | 13.142 | 1.3731 | 4.2577 | - | - | - | - |
| vic_no_mig_admix_early | -462.88 | 937.76 | 343.25 | 54.8 | 0.3137 | 1.9374 | 8.853 | 0.0164 | 0.4957 | 0.981 | - | - | - |
| sym_mig_twoepoch | -463.09 | 938.18 | 286.24 | 80.5 | 0.1662 | 0.2634 | 0.2287 | 8.0011 | 4.0049 | 0.641 | - | - | - |
| vic_no_mig_admix_late | -463.68 | 939.36 | 350.23 | 54.38 | 0.3303 | 0.9671 | 1.058 | 0.0168 | 0.4913 | 0.0192 | - | - | - |
| asym_mig_size | -463.07 | 942.14 | 260.74 | 32.58 | 0.1928 | 0.0216 | 0.2387 | 1.237 | 7.3603 | 1.0473 | 1.0093 | 23.4345 | - |
| sec_contact_asym_mig | -465.22 | 942.44 | 273.04 | 23.48 | 0.4645 | 1.5102 | 4.3428 | 1.2911 | 0.5749 | 20.6707 | - | - | - |
| asym_mig_twoepoch | -463.56 | 943.12 | 260.32 | 32.23 | 0.1057 | 1.4807 | 24.6382 | 0.1632 | 17.648 | 0.6088 | 0.5229 | 25.8214 | - |
| vic_sec_contact_asym_mig | -466.56 | 949.12 | 304.45 | 44.57 | 0.7744 | 0.9143 | 0.3918 | 3.7959 | 5.1897 | 2.2096 | 0.796 | 0.4988 | - |
| founder_asym | -467.57 | 949.14 | 273.67 | 46.93 | 0.4768 | 2.1528 | 0.7995 | 7.1827 | 1.9197 | 9.7941 | 0.4703 | - | - |
| sec_contact_sym_mig_three_epoch | -470.36 | 952.72 | 322.38 | 7.03 | 2.3634 | 2.6791 | 0.8014 | 15.6807 | 3.7049 | 0.0262 | - | - | - |
| vic_no_mig | -474.2 | 958.4 | 376.5 | 52.07 | 0.4258 | 14.8029 | 2.244 | 0.019 | 0.3618 | - | - | - | - |
| anc_sym_mig | -475.35 | 960.7 | 319.32 | 13.7 | 1.4452 | 1.8091 | 1.3291 | 17.5831 | 0.0103 | - | - | - | - |
| founder_sym | -474.8 | 961.6 | 323.39 | 4.64 | 10.3828 | 0.0839 | 2.8568 | 0.422 | 1.114 | 0.4995 | - | - | - |
| anc_asym_mig_size | -476.84 | 969.68 | 391.59 | 6.95 | 5.8338 | 1.6354 | 0.9449 | 2.5627 | 0.5301 | 1.4975 | 23.0606 | 0.0595 | - |
| vic_anc_asym_mig | -480.89 | 977.78 | 346.06 | 27.82 | 1.6159 | 0.5956 | 0.4705 | 2.8591 | 3.1838 | 4.4063 | 0.0103 | 0.4967 | - |
| sec_contact_asym_mig_three_epoch | -482.05 | 978.1 | 367.25 | 14.34 | 0.7493 | 2.8106 | 19.7435 | 0.7021 | 3.9548 | 0.0539 | 0.3573 | - | - |
| founder_nomig_admix_two_epoch | -483.39 | 980.78 | 397.12 | 3.81 | 13.1324 | 1.9635 | 2.0811 | 0.2944 | 4.5421 | 0.4916 | 0.5534 | - | - |
| anc_asym_mig | -485.42 | 982.84 | 390.78 | 13.32 | 0.6568 | 2.9831 | 8.6432 | 2.1178 | 18.0841 | 0.0558 | - | - | - |
| founder_nomig | -489.06 | 988.12 | 417.74 | 3.13 | 15.5765 | 0.2472 | 1.7908 | 0.4605 | 0.4986 | - | - | - | - |
| founder_nomig_admix_late | -488.1 | 988.2 | 423.03 | 13.92 | 3.5196 | 1.8622 | 0.3334 | 0.0999 | 0.4969 | 0.0284 | - | - | - |
| sec_contact_asym_mig_size_three_epoch | -485.69 | 989.38 | 339.02 | 31.19 | 0.523 | 0.0652 | 0.5077 | 1.1734 | 3.0128 | 2.0254 | 0.3257 | 1.708 | 0.0101 |
| founder_nomig_admix_early | -488.97 | 989.94 | 417.44 | 9.66 | 5.0677 | 0.8197 | 0.4709 | 0.1352 | 0.4993 | 0.1072 | - | - | - |
| vic_two_epoch_admix | -507.4 | 1028.8 | 485.43 | 38 | 1.2294 | 0.7103 | 2.4985 | 3.6091 | 0.0131 | 0.0617 | 0.9795 | - | - |
| no_mig_size | -519.45 | 1050.9 | 507.65 | 42.96 | 0.2095 | 0.4013 | 5.9345 | 1.2277 | 0.0276 | 0.0232 | - | - | - |
